# Meta reinforcement learning for robust quadruped locomotion under joint failures

**DOI:** 10.1371/journal.pone.0361099

**Published:** 2026-10-16

**Authors:** Likai Wu, Chee-Onn Chow, Wei Ru Wong, Joon Huang Chuah, Jeevan Kanesan, Meina Zhang

**Affiliations:** 1 Department of Electrical Engineering, Faculty of Engineering, Universiti Malaya, Kuala Lumpur, Malaysia; 2 School of Electrical Engineering, Shandong Huayu University of Technology, Dezhou, China; 3 Department of Naval Architecture and Ocean Engineering, Pusan National University, Busan, South Korea; Zhejiang University, CHINA

## Abstract

Joint failures can disrupt gait coordination, velocity tracking, and body stability in quadruped robots by reducing the available control authority. This study investigates faults in which joints remain locked at fixed angles and presents Adjudicate Reconfiguration and Evolving Regularization Meta Reinforcement Learning (ARER-MetaRL). The framework combines task inference from context, modular routing tailored to each task through an Adjudicate Reconfiguration (AR) network, and Evolving Gradient Regularization (ER) to support rapid adaptation. Tasks differ in the identities and locking angles of the faulty joints, the number of simultaneous faults, the commanded motion direction, and the terrain. Experiments in NVIDIA Isaac Sim cover all 12 actuated joints, with 0–3 stuck joints during meta-training and severity tests involving up to 6 simultaneously stuck joints. ARER-MetaRL achieves higher Mean Episode Linear Velocity Tracking Reward (MELV) and Mean Episode Roll–Pitch Tracking Reward (MERP) than single-task reinforcement learning, ablated variants, and the evaluated learning and adaptation baselines. Cumulative rewards further demonstrate the benefit of combining the proposed components. In the study of fault severity, ARER-MetaRL achieves MELV/MERP values of 0.86/0.84 with one stuck joint and 0.55/0.54 with six stuck joints, compared with 0.21/0.18 and 0.05/0.03, respectively, for the single-task baseline. These results show improved adaptation across different configurations of locked joints and terrain conditions.

## 1. Introduction

Quadruped robots can traverse unstructured environments in which wheeled platforms have limited mobility. A recent review organizes research on AI for quadruped robots around locomotion capabilities, validation maturity, and deployment challenges, including robustness, transfer from simulation to reality, and fault diagnosis [1]. Traversing difficult terrain requires coordinated actuation across multiple joints. A joint fault can change a leg’s reachable workspace, disrupt the timing of ground contact, redistribute loads among the remaining limbs, and introduce coupled errors in body velocity, roll, and pitch. Actuator faults include joint locking, complete power loss, torque degradation, intermittent actuation, and freely swinging joints. When a joint becomes locked at a fixed angle, the remaining joints must compensate for the loss of an actively controlled degree of freedom. This study focuses on joint locking.

Adaptation to joint locking faults is challenging because the resulting dynamics depend on the faulty joint’s location and locking angle, the number of simultaneous faults, the commanded motion, and the terrain. A controller trained for a hip fault in a front leg may therefore perform poorly when a knee in a rear leg locks or when two joints fail simultaneously. Designing a separate recovery controller for every configuration is impractical. Conversely, training a single policy across all configurations can introduce conflicting task gradients and limit specialization. An effective adaptation method must use a short interaction history to infer the current fault condition and adjust the policy with limited additional data.

Traditional fault-tolerant control (FTC) methods rely primarily on analytical gait planning, kinematic constraints, analysis of residual mobility, and control reconfiguration [2–6]. These methods provide interpretable solutions for specified joint locking conditions. Xi et al. planned feasible joint trajectories for hexapod robots with partially locked active joints while preserving the original gait sequence [7]. Recent model-based work also addresses actuator faults: Anjum et al. developed an adaptive fixed-time sliding-mode controller for manipulator trajectory tracking under actuator gain and bias faults [8]. For quadruped locomotion with locked joints, changes in the fault location, locking angle, or number of failed joints can require a new feasibility analysis, controller redesign, or parameter adjustment. Extending an explicit model to a broad range of combined fault and terrain conditions is also difficult.

Learning-based methods reduce the need to specify recovery rules manually. Reinforcement learning (RL), teacher–student architectures, and adaptive controllers have improved locomotion under actuator degradation and joint failures [9–11]. Recent work has expanded the range of faults that can be addressed. Hou et al. developed a hierarchical multitask controller for joint power loss and locking, including simultaneous faults in two joints of one leg [12]. FT-CPG combines central pattern generators with RL for locomotion under multiple joint failures [10], while learning from small samples has been used for fault identification and control [11]. Hybrid deep neural networks combining convolutional neural networks, gated recurrent units, and attention mechanisms have also been used to identify quadruped faults from sensor data [13]. Another approach combines proprioceptive history with a reliability reward to adapt locomotion to an unknown motor failure [14].

Predictive feedback provides another route to fault adaptation. Fu et al. combined contrastive learning with a forward prediction model and fed state prediction errors into the controller, reporting zero-shot adaptation to locked joints [15].

Complementary approaches improve locomotion through structured gait generation and control that accounts for uncertainty. Wu et al. combined central pattern generators with residual coordination among agents assigned to individual legs, enabling terrain adaptation on an inexpensive quadruped [16]. Wu used multimodal intent inference, intent confidence, and locomotion risk to adjust human–robot control authority during guided locomotion on uneven terrain [17]. These approaches address gait coordination and interaction uncertainty. The present study focuses on rapid policy adaptation when joint locking changes the available actuation.

Meta reinforcement learning (Meta-RL) supports rapid adaptation by learning an initialization or a latent task representation that can be updated using a short interaction history. Meta-RL based on context has been applied to quadruped locomotion with locked motors, demonstrating task inference and rapid policy adjustment [18]. Recent studies also investigate multitask reinforcement learning for robotic manipulator control [19] and Evolving Gradient Regularization for Meta-RL [20]. These studies provide complementary context for policy organization across tasks and regularization during adaptation. Two issues motivate the present study. First, gradients from tasks involving different legs, joint types, or combinations of locked joints can interfere with one another. Second, a small adaptation dataset can lead to unstable inner-loop updates and poor generalization to combinations of faults and terrain outside the meta-training distribution. We address these issues jointly through modular routing tailored to each task and regularization of the adaptation process.

We develop Adjudicate Reconfiguration and Evolving Regularization Meta Reinforcement Learning (ARER-MetaRL) for this purpose. The AR network uses a context embedding to route information through policy submodules relevant to the current task. ER smooths the gradients in the inner loop and uses parameter perturbations to guide the meta-update toward robust parameter regions. We evaluate the framework in NVIDIA Isaac Sim across joint locations, locking angles, numbers of simultaneous faults, motion directions, and terrain conditions.

The main contributions are as follows:

1. A Meta-RL formulation based on context captures variation in the identity of the faulty joints, locking angle, number of simultaneous faults, motion direction, and terrain condition.
2. An Adjudicate Reconfiguration network routes information through policy submodules selected for each task to reduce gradient interference across different configurations of locked joints.
3. Evolving Gradient Regularization stabilizes adaptation in the inner loop with limited data and supports generalization to unseen combinations of faults and terrain.
4. Isaac Sim experiments cover all 12 actuated joints, meta-training with 0–3 stuck joints, and severity tests with up to 6 stuck joints. Ablation studies, baseline comparisons, and evaluations of diagonal motion and locomotion on rough terrain quantify performance using MELV, MERP, and cumulative reward.

### 1.1. Structure of the paper

The remainder of this paper is organized as follows. Section 2 reviews fault-tolerant control for legged robots, learning-based locomotion, and Meta-RL. Section 3 presents the problem formulation, network architecture, and training procedure. Section 4 describes the experimental setup and reports the ablation studies, baseline comparisons, and evaluations across motion and terrain conditions. Section 5 summarizes the findings and outlines future work.

## 2. Related work

### 2.1. Model-based fault-tolerant control for legged robots

Traditional FTC methods for legged robots use kinematic analysis, geometric constraints, and predefined gait patterns. Early quadruped studies developed fault-tolerant gaits and kinematic constraints for failures involving locked joints [2,3]. These studies established an analytical basis for locomotion recovery under specified joint failure conditions.

Subsequent studies considered more severe faults. Methods based on GF set theory analyze residual mobility and generate omnidirectional gaits for quadruped robots with one or two locked legs [5,6]. Universal gait planning for hexapod robots with partially locked active joints preserves the original gait sequence while optimizing feasible joint trajectories [7]. For quadruped robots with multiple faulty actuators, constrained optimization exploits the remaining degrees of freedom to reduce body displacement [4].

Other model-based studies have investigated compliant robot designs, control reconfiguration, and disturbance rejection. Gor et al. developed a compliant legged quadruped and experimentally evaluated control strategies for joint locking faults [21]. Recovery strategies inspired by infant crawling reconfigure the robot’s posture after failures leave joints swinging freely [22]. Linear active disturbance rejection control with multiple extended state observers, as well as motion planning combined with whole-body control, addresses disturbances and posture regulation under actuator faults [23,24]. These methods offer interpretable solutions for specified conditions, although extending their analytical models and designs tailored to particular tasks to new combinations of faults remains challenging.

### 2.2. Learning-based methods for fault-tolerant control

Learning-based methods train policies across a range of dynamics and fault conditions, reducing the need for manually designed recovery rules. Early work randomized physical parameters during training to improve robustness to uncertain robot dynamics [25]. Adaptive neural and fuzzy control approaches addressed actuator faults and model uncertainty without requiring exact dynamic models [26]. More recent methods for fault diagnosis combine convolutional neural networks, gated recurrent units, and attention mechanisms to identify quadruped faults from sensor data [13].

Deep RL has also been used to learn recovery policies. FT-Net combines a unified policy, a neural adaptor, and rewards that encourage balance to recover from motor failures [9]. FT-CPG integrates central pattern generators with RL to achieve locomotion under multiple joint failures [10]. Studies of actuator degradation and joint locking have explored teacher–student architectures and multitask learning, including transfer from simulation to physical robots [12,27,28]. Another approach combines proprioceptive history with a reliability reward to adapt to an unknown motor failure [14].

The baselines considered in this study include the Fast Adaptation Dynamics Model (FADM), Gradient-Based Adaptive Learner (GrBAL), Decision Transformer (DT), and Randomized Ensembled Double Q-Learning (REDQ). FADM combines an ensemble dynamics model with model predictive planning for damage recovery [29]. GrBAL uses meta learning to initialize a dynamics model for online adaptation through gradient updates using recent transitions [30]. DT treats control as sequence modeling conditioned on the desired return [31]. REDQ uses an ensemble of critics to improve sample efficiency and value estimation [32]. These methods provide complementary baselines for evaluating the proposed combination of routing tailored to each task and regularized adaptation.

### 2.3. Meta reinforcement learning for robotic fault-tolerant control

Meta-RL learns policies, initializations, or representations that can adapt rapidly to new tasks. Approaches based on context and probabilistic inference use interaction histories to estimate task representations or dynamics and support adaptation under changing conditions. Relevant studies investigate variational information bottleneck regularization, Bayesian nonparametric task inference, nonstationary environments, and probabilistic model-based adaptation [33–36]. Other studies investigate collaborative generator and worker networks, Adjudicate Reconfiguration networks, and Evolving Gradient Regularization to improve flexibility and adaptation [19,20,37].

For quadruped fault tolerance, Meta-RL based on context combines a context encoder, a reference action generator, and a selection module to adapt locomotion policies to locked motors [18]. This approach establishes task inference as a practical mechanism for fault adaptation. The present study examines how modular routing tailored to each task and regularized updates in the inner loop can be combined to handle a wider range of configurations of locked joints with limited adaptation data.

### 2.4. Comparative summary and research gap

Table 1 summarizes the fault models and evaluation settings of representative studies. Analytical methods characterize gait feasibility or stability under specified joint and leg constraints. Learning-based methods cover a broader range of faults and, in several cases, have been evaluated on physical robots. The most closely related study of Meta-RL based on context evaluates locomotion with locked motors in both simulation and hardware [18]. ARER-MetaRL combines modular routing tailored to each task with regularized fast adaptation and evaluates this combination across joint locations, locking angles, numbers of simultaneous faults, motion directions, and terrain conditions.

**Table 1:** Comparison of representative fault-tolerant locomotion studies. The table summarizes the reported fault models and evaluation settings. Performance measures vary across studies because they use different robots, tasks, and metrics.

Study	Method	Fault scope	Validation and evaluation scope
Yang [2]	Analytical fault-tolerant gait	One locked joint	Quadruped gait analysis; recovery is designed for a condition involving a single locked joint.
Chen et al. [4]	Kinematic analysis and constrained motion planning	Partially faulty actuators	Optimization in simulation exploits the residual degrees of freedom under multiple actuator constraints.
Chen et al. [5]	Gait planning using GF sets and residual mobility	One locked leg	Simulation; omnidirectional walking is designed while maintaining a nonzero static stability margin.
Chen et al. [6]	Fault-tolerant gait planning using GF sets	Two locked legs	Simulation; extends analysis of residual mobility from one to two locked legs.
Luo et al. (FT-Net) [9]	Model-free RL with a neural adaptor	Partial and complete motor failures	Simulation and hardware; a unified policy switches to three-legged locomotion after complete motor failure.
Zhang et al. (FT-CPG) [10]	Model-free RL guided by CPGs	Concurrent locked or powerless joints	Simulation and Unitree Go1 experiments; evaluates multiple faulty joints within a leg and zero-shot transfer from simulation to reality.
Chen et al. [18]	Meta-RL based on context with RAG and selection	Locked motors	Simulation and physical robot experiments; uses short context histories for rapid adaptation.
**ARER-MetaRL (this study)**	Context encoder, AR modular routing, and ER	Joints locked at fixed angles	Isaac Sim; 12 candidate joints, 0–3 locks in training, 1–6 locks in severity testing, rollouts of 200 steps, 10 runs per setting, and 5 updates in the inner loop.

## 3. Methodology

This section presents the proposed Adjudicate Reconfiguration and Evolving Regularization Meta Reinforcement Learning (ARER-MetaRL) framework. It addresses two challenges identified in Section 2: interference between tasks with different configurations of locked joints and unstable adaptation when online data are limited.

Fig 1 shows the ARER-MetaRL workflow. Each combination of locked joints and terrain conditions is represented as a Markov decision process for a distinct task. A context encoder infers a latent task representation from a short rollout, and the Adjudicate Reconfiguration network uses this representation to assign weights to policy submodules. Evolving Gradient Regularization smooths updates in the inner loop and guides the meta-update toward parameter regions with higher returns under perturbation.

**Fig 1:**
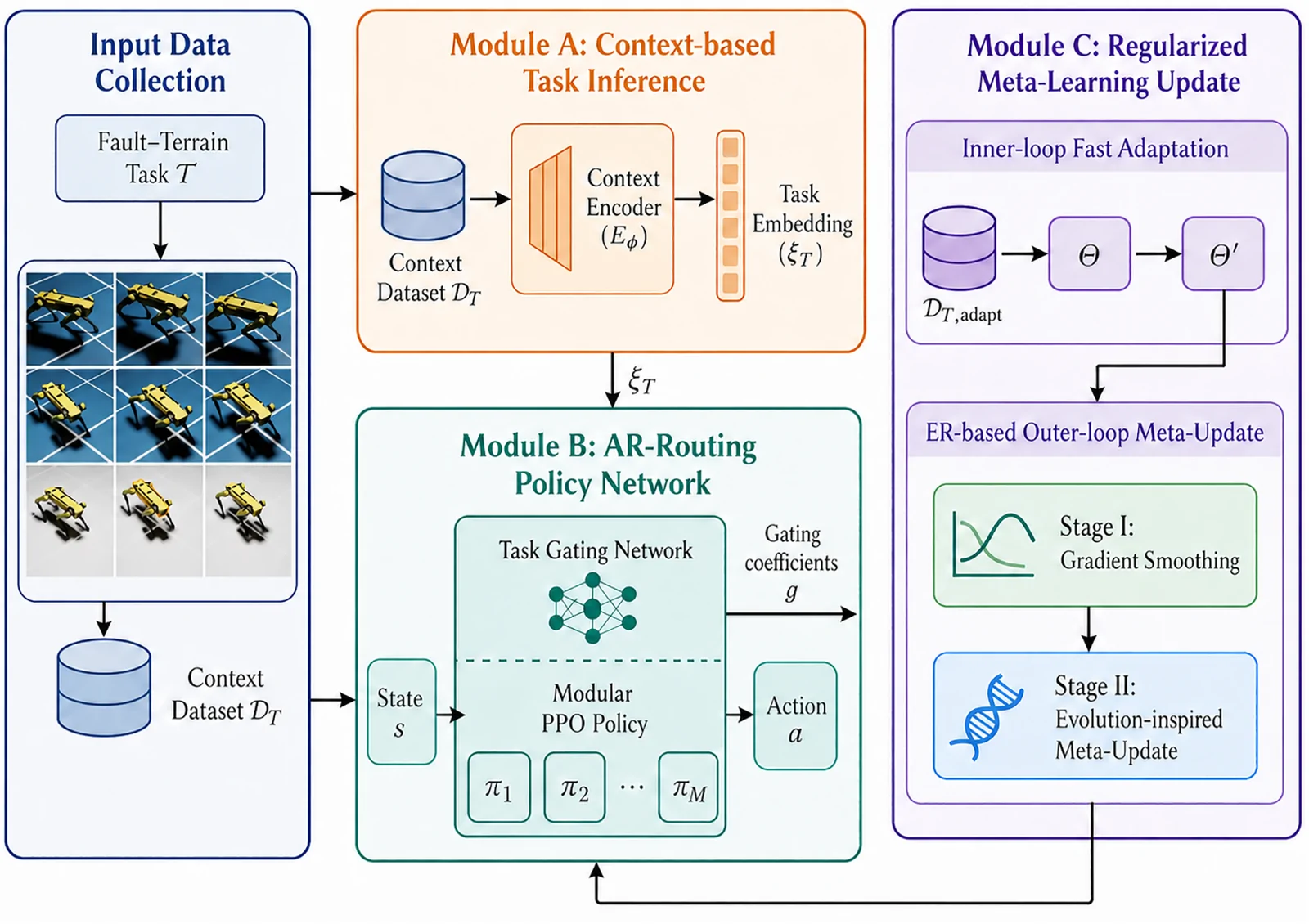
Overview of the proposed ARER-MetaRL framework. A short rollout is encoded as a task embedding that determines AR routing weights for fast adaptation regularized by ER. The robot images in the “Input Data Collection” panel are screenshots captured by the authors in NVIDIA Isaac Sim.

### 3.1. Problem formulation

Quadruped locomotion under joint locking faults and varying terrain conditions is formulated as a distribution of Markov decision processes (MDPs), one for each task. Let *T* denote a task index corresponding to a particular configuration of locked joints and terrain condition. The MDP for task *T* is defined as


$$\begin{array}{c} {ℳ}_{T}=\langle 𝒮,𝒜,p_{T},r_{T},\gamma \rangle , \end{array}$$ (1)

where 𝒮 and 𝒜 are the state and action spaces, $p_{T}(s^{\prime }|s,a)$ is the transition function for the task, $r_{T}(s,a)$ is the reward function for the task, and γ∈(0,1) is the discount factor. Thus, *T* identifies the fault and terrain condition, and ${ℳ}_{T}$ denotes its MDP. Tasks drawn from *p*(*T*) are divided into a training set *T*_train_ and a test set *T*_test_.

The Meta-RL objective is to learn meta-parameters Θ that support rapid adaptation to an unseen task T~p(T) using limited data:


$$\begin{array}{c} \max_{\Theta }{𝔼}_{T\sim p(T)}\left[{𝔼}_{\tau \sim \pi_{\Theta_{T}^{\prime }}}\sum_{t=0}^{\infty }\gamma^{t}r_{T}(s_{t},a_{t})\right], \end{array}$$ (2)

where $\Theta_{T}^{\prime }$ denotes the parameters obtained after a short adaptation phase for the task.

### 3.2. Meta-RL framework

The framework uses gradient-based Meta-RL with proximal policy optimization (PPO) as the base algorithm. For each task *T*, a small context dataset $D_{T}=\{(s_{i},a_{i},r_{i},s_{i}^{\prime }){\}}_{i=1}^{N}$ is collected, and the policy parameters are adapted through *K* gradient updates in the inner loop:


$$\begin{array}{c} \Theta_{T}^{\left(k+1\right)}=\Theta_{T}^{\left(k\right)}-\alpha \nabla_{\Theta_{T}^{\left(k\right)}}L_{T}(\Theta_{T}^{\left(k\right)};D_{T}), \end{array}$$ (3)

A meta-update in the outer loop then aggregates gradients across tasks. The inner loop enables rapid policy adjustment using limited online data.

### 3.3. Context encoder for task inference

Because the task identity is unknown at deployment, a context encoder $E_{\phi }$ maps the context dataset $D_{T}$ to a task embedding:


$$\begin{array}{c} \xi_{T}=E_{\phi }(D_{T}). \end{array}$$ (4)

The embedding $\xi_{T}$ conditions the AR network to assign weights to its submodules for each task, allowing the policy to use information about fault dynamics inferred from a short interaction history.

### 3.4. Adjudicate reconfiguration (AR) network

To mitigate **gradient interference** between tasks with different configurations of locked joints, each hidden layer of the policy network is divided into *M* parallel submodules:


$$\begin{array}{c} u_{m}^{\ell }=f_{m}^{\ell }(h^{\ell }),\;m=1,...,M, \end{array}$$ (5)

A gating network conditioned on the task produces the coefficients


$$\begin{array}{c} g^{\ell }=\text{softmax}(W^{\ell }\xi_{T}+b^{\ell }), \end{array}$$ (6)

and the layer output is computed as


$$\begin{array}{c} h^{\ell +1}=\sum_{m=1}^{M}g_{m}^{\ell }u_{m}^{\ell }. \end{array}$$ (7)

This weighted combination emphasizes submodules relevant to the current task and is designed to reduce interference between different configurations of locked joints.

### 3.5. Evolving gradient regularization (ER)

ER is designed to improve adaptation stability and out-of-distribution (OOD) generalization through two stages:

**Stage I: Gradient Smoothing in the Inner Loop**


$$\begin{array}{c} {\tilde{g}}_{k}=\rho {\tilde{g}}_{k-1}+(1-\rho )g_{k}, \end{array}$$ (8)

where $g_{k}=\nabla_{\Theta_{T}^{\left(k\right)}}L_{T}$.

**Stage II: Meta-Update Inspired by Evolution**


$$\begin{array}{c} \Theta_{T,e}^{\prime }=\Theta_{T}^{\prime }+\sigma \epsilon_{e},\;\epsilon_{e}\sim 𝒩(0,I), \end{array}$$ (9)

Meta-gradients weighted by fitness then guide learning toward parameter regions that yield higher returns under perturbation, with the aim of improving generalization to unseen configurations of locked joints and terrain conditions.

### 3.6. State, action, and reward design

- **State**: base orientation, linear and angular velocities, joint positions and velocities, and contact indicators.
- **Action**: residual joint position commands relative to the nominal posture, tracked by PD controllers.
- **Reward**: a combination of terms for velocity tracking, posture stability, energy efficiency, foot clearance, and fall penalties.

The same reward formulation is used across tasks, providing a consistent objective for routing and adaptation tailored to each task.

### 3.7. Training procedure

Algorithm 1 summarizes the meta-training procedure. For each task sampled from the training distribution, a short rollout populates the context buffer. The context encoder produces a task embedding, and the AR network uses it to determine the routing weights. Adaptation in the inner loop uses gradients smoothed by ER, while the update in the outer loop aggregates meta-gradients across tasks using fitness weights.

**Algorithm 1. Meta-Training with AR and ER.**

**Require:** Task distribution *p*(*T*), training set *T*_train_, meta-parameters Θ, context encoder parameters ϕ, PPO hyperparameters, steps in the inner loop *K*, ER parameters.

**Ensure:** Trained meta-parameters Θ and context encoder $E_{\phi }$.

1: Initialize Θ, $E_{\phi }$, and AR gating parameters.

2: **for** $n=1,2,...,N_{meta}$ **do**

3:    Sample a minibatch of tasks $ℬ=\{T_{i}{\}}_{i=1}^{B}$ from *T*_train_.

4:   𝒢←0.

5:   **for** each task T∈ℬ **do**

6:     Collect a short rollout using $\pi_{\Theta }$ to build context $D_{T}$.

7:     Infer task embedding $\xi_{T}\leftarrow E_{\phi }(D_{T})$.

8:     Compute AR gates $g_{T}^{\ell }\leftarrow softmax(W^{\ell }\xi_{T}+b^{\ell })$.

9:     Set $\Theta_{T}^{\left(0\right)}\leftarrow \Theta$ and ${\tilde{g}}_{-1}\leftarrow 0$.

10:     **for** k=0,1,...,K−1 **do**

11:       Compute PPO loss ${ℒ}_{T}(\Theta_{T}^{\left(k\right)})$.

12:       Compute gradient $g_{k}\leftarrow \nabla_{\Theta_{T}^{\left(k\right)}}{ℒ}_{T}$.

13:       Smooth gradient ${\tilde{g}}_{k}\leftarrow \rho {\tilde{g}}_{k-1}+(1-\rho )g_{k}$.

14:       Update $\Theta_{T}^{\left(k+1\right)}\leftarrow \Theta_{T}^{\left(k\right)}-\alpha {\tilde{g}}_{k}$.

15:     **end for**

16:     $\Theta_{T}^{\prime }\leftarrow \Theta_{T}^{\left(K\right)}$.

17:     **for** e=1,2,...,E **do**

18:       Sample perturbation $\epsilon_{e}\sim 𝒩(0,I)$.

19:       $\Theta_{T,e}^{\prime }\leftarrow \Theta_{T}^{\prime }+\sigma \epsilon_{e}$.

20:       Evaluate return $F_{e}\leftarrow J_{T}(\Theta_{T,e}^{\prime })$.

21:     **end for**

22:     Compute $w_{e}\leftarrow \frac{\exp(\eta F_{e})}{\sum_{j=1}^{E}\exp(\eta F_{j})}$.

23:     Compute $\nabla_{\Theta }^{ER}(T)\leftarrow \sum_{e=1}^{E}w_{e}\nabla_{\Theta }[-J_{T}(\Theta_{T,e}^{\prime })]$.

24:     $𝒢\leftarrow 𝒢+\nabla_{\Theta }^{ER}(T)$.

25:    **end for**

26:    Update $\Theta \leftarrow \Theta -\beta B^{-1}𝒢$.

27: **end for**

Algorithm 2 summarizes the fast adaptation procedure used at deployment. For an unseen combination of fault and terrain conditions, the policy first performs a short probing rollout to infer a task embedding. The corresponding AR routing weights are then held fixed while a small number of gradient updates smoothed by ER are applied. The resulting policy is used for task execution.

**Algorithm 2. Fast Adaptation with AR and ER**

**Require:** Meta-trained parameters Θ, context encoder $E_{\phi }$, steps in the inner loop *K*, adaptation step size α.

**Ensure:** Parameters adapted to the task $\Theta_{T}^{\prime }$.

1: Initialize empty context buffer D←∅.

2: Execute a short probing rollout using $\pi_{\Theta }$ and collect transitions into *D*.

3: Infer task embedding $\xi \leftarrow E_{\phi }(D)$.

4: Compute AR gates $g^{\ell }\leftarrow softmax(W^{\ell }\xi +b^{\ell })$ for all layers.

5: Set $\Theta^{\left(0\right)}\leftarrow \Theta$ and ${\tilde{g}}_{-1}\leftarrow 0$.

6: **for** k=0,1,...,K−1 **do**

7:   Compute PPO adaptation loss $ℒ(\Theta^{\left(k\right)};D)$.

8:   Compute gradient $g_{k}\leftarrow \nabla_{\Theta^{\left(k\right)}}ℒ$.

9:   Smooth gradient ${\tilde{g}}_{k}\leftarrow \rho {\tilde{g}}_{k-1}+(1-\rho )g_{k}$.

10: Update $\Theta^{\left(k+1\right)}\leftarrow \Theta^{\left(k\right)}-\alpha {\tilde{g}}_{k}$.

11: **end for**

12: Output adapted parameters $\Theta_{T}^{\prime }\leftarrow \Theta^{\left(K\right)}$.

13: Deploy policy $\pi_{\Theta_{T}^{\prime }}$ with fixed AR gates.

## 4. Experiments

### 4.1. Experimental setup

#### 4.1.1. Simulation platform.

All experiments are conducted in NVIDIA Omniverse Isaac Sim [38] using a quadruped model with 12 degrees of freedom. The PhysX rigid-body simulation runs at 1000 Hz, and the control policy runs at 50 Hz. Each action is held constant for 20 consecutive simulation steps. The simulator provides reproducible contact and terrain conditions and allows controlled variation of terrain geometry, friction, and joint constraints for systematic comparisons across fault configurations.

#### 4.1.2. Joint locking fault configuration.

A fault that locks a joint at a fixed angle is simulated by overriding the command to a selected actuated joint and holding it at a constant angle during locomotion. Each fault configuration specifies the affected joints, their sampled stuck angles, and the number of joints that are simultaneously locked. This model represents a locked joint; reduced actuator torque and freely swinging joints are considered in future work.

- **Candidate joints:** All 12 actuated leg joints, including hip abduction/adduction (HAA), hip flexion/extension (HFE), and knee flexion/extension (KFE) joints on each leg.
- **Number of stuck joints during meta-training:** $N_{f}^{train}\in \{0,1,2,3\}$.
- **Out-of-distribution severity evaluation:** $N_{f}^{test}\in \{1,2,3,4,5,6\}$. Configurations with four to six concurrently stuck joints are used only for the severity study and are not included in meta-training.
- **Stuck angle ranges by joint type:** The stuck angle for each faulty joint is sampled according to its joint type:
  – HAA joints: $\theta_{\text{stuck}}\in [-0.25,\;0.25]$ rad
  – HFE joints: $\theta_{\text{stuck}}\in [-0.60,\;0.60]$ rad
  – KFE joints: $\theta_{\text{stuck}}\in [0.30,\;1.20]$ rad

During meta-training, a curriculum with three stages progressively scales the stuck angle ranges for each joint type:

- Stage 1: 30% of the full angle ranges
- Stage 2: 60% of the full angle ranges
- Stage 3: 100% of the full angle ranges

Training proceeds sequentially through these stages. Evaluation uses the full stuck angle ranges for all fault configurations. Table 2 summarizes the ranges and curriculum stages for each joint type.

**Table 2:** Stuck angle ranges and curriculum stages for each joint type during training and evaluation.

Joint Type	Stuck Angle Range (rad)	Curriculum Scaling	Evaluation Scaling
Hip Abduction/Adduction (HAA)	[−0.25,0.25]	30%→60%→100%	100%
Hip Flexion/Extension (HFE)	[−0.60,0.60]	30%→60%→100%	100%
Knee Flexion/Extension (KFE)	[0.30, 1.20]	30%→60%→100%	100%

The terrain configurations used for training and evaluation are summarized in Table 3.

**Table 3:** Terrain configurations used for training and evaluation.

Terrain Type	Key Parameters	Training	Evaluation
Flat ground	Friction coefficient μ=1.0	✓	✓
Surface with low friction	Friction coefficient μ=0.3	✓	✓
Inclined slope	Slope angle $\alpha \in [5^{\circ },\;15^{\circ }]$	✓	✓
Uneven terrain	Height variation Δh∈[0.02,0.06] m	✓	✓
Random obstacles	Obstacle height h∈[0.05,0.15] m	✓	✓

#### 4.1.3. Algorithm implementation and hyperparameters.

The framework uses PPO as the base reinforcement learning algorithm. The policy and value networks are implemented as multilayer perceptrons, each with two hidden layers of 256 units and ReLU activation functions.

The context encoder uses an architecture invariant to permutations with an embedding dimension of $d_{\xi }=32$. During meta-training, a context buffer containing $K_{c}=200$ time steps is collected for each task.

Fast adaptation uses *K* = 5 gradient updates in the inner loop with a step size of $\alpha =3\times 10^{-4}$. The learning rate in the outer loop is $\beta =1\times 10^{-4}$.

PPO uses a clipping ratio of 0.2, a discount factor of γ=0.99, and a generalized advantage estimation parameter of λ=0.95. Each policy update comprises 10 epochs with a minibatch size of 64.

For the ER module, we sample *E* = 8 perturbed parameter vectors using Gaussian noise ϵ~𝒩(0,I) with a noise scale of σ=0.01. Aggregation by fitness uses softmax weights with a scaling coefficient of η=1.0.

The hyperparameters are held fixed across fault configurations and terrain conditions to support consistent comparisons.

The main hyperparameters used in all experiments are summarized in Table 4.

**Table 4:** Hyperparameters used in all experiments.

Hyperparameter	Value
Simulation platform	Isaac Sim (PhysX)
Physics update frequency	1000 Hz
Policy control frequency	50 Hz
Action repeat	20 steps
Robot degrees of freedom	12
Episode length	200 steps
Context buffer size $K_{c}$	200 steps
Policy / value network	2×256 (ReLU)
Context embedding dimension $d_{\xi }$	32
Adaptation steps in the inner loop *K*	5
Learning rate in the inner loop α	$3\times 10^{-4}$
Learning rate in the outer loop β	$1\times 10^{-4}$
PPO clipping ratio	0.2
Discount factor γ	0.99
GAE parameter λ	0.95
PPO epochs per update	10
Minibatch size	64
ER perturbation samples *E*	8
Noise scale σ	0.01
Softmax scaling coefficient η	1.0

#### 4.1.4. Evaluation metrics.

Fault-tolerant locomotion is evaluated using three metrics based on rewards for velocity tracking, posture regulation, and actuation effort. All metrics are computed over rollouts of 200 steps and averaged across 10 independent runs for each setting. Higher values indicate better performance.

**1) Velocity Tracking Reward (MELV).** Velocity tracking performance is quantified using the *Mean Episode Linear Velocity Tracking Reward (MELV)*, defined as the average over an episode of the velocity tracking reward *r*_1_ at each step:


$$\begin{array}{c} \text{MELV}=\frac{1}{T}\sum_{t=1}^{T}r_{1}(t), \end{array}$$ (10)

where *T* = 200 is the episode length and *r*_1_(*t*) is the controller’s exponential velocity tracking reward, computed from the difference between the target forward velocity $v_{x}^{\text{ref}}$ and the measured base velocity $v_{x}$. Smaller errors yield higher rewards, so a higher MELV indicates more accurate velocity tracking under joint locking faults.

**2) Posture Stability Reward (MERP).** Posture regulation is evaluated using the *Mean Episode Roll–Pitch Tracking Reward (MERP)*, defined as the average over an episode of the posture reward *r*_2_ at each step:


$$\begin{array}{c} \text{MERP}=\frac{1}{T}\sum_{t=1}^{T}r_{2}(t), \end{array}$$ (11)

where *r*_2_(*t*) penalizes deviations in the robot’s roll and pitch angles from the desired upright posture. A higher MERP indicates better regulation of body roll and pitch during locomotion under joint locking faults.

**3) Energy Efficiency Reward.** Actuation effort is assessed using the reward term *r*_4_ for energy efficiency, which penalizes excessive joint torque effort:


$$\begin{array}{c} R_{E}=\frac{1}{T}\sum_{t=1}^{T}r_{4}(t), \end{array}$$ (12)

where *r*_4_(*t*) is computed from joint torque effort. Higher values of $R_{E}$ indicate lower actuation effort under this reward definition.

### 4.2. Ablation study

We evaluate the contribution of each component through an ablation study with 0–3 simultaneously stuck joints. The comparison includes Single-task RL, ARER-MetaRL without the context encoder, ARER-MetaRL without the AR network, ARER-MetaRL without ER, and the full ARER-MetaRL model. Higher values of these reward metrics indicate better performance.

All methods are trained and evaluated under the experimental settings described in Section 4.1. Performance is assessed using MELV, MERP, cumulative reward, and a qualitative comparison of foot trajectories.

Fig 2 shows MELV and MERP as the number of simultaneously stuck joints increases. Case 1 and Case 2 are two representative samples from the evaluation task distribution, with different joint locations and stuck angles. The Single-task RL baseline declines in both metrics as fault severity increases, indicating poorer velocity tracking and posture regulation as fewer joints remain available for control.

**Fig 2:**
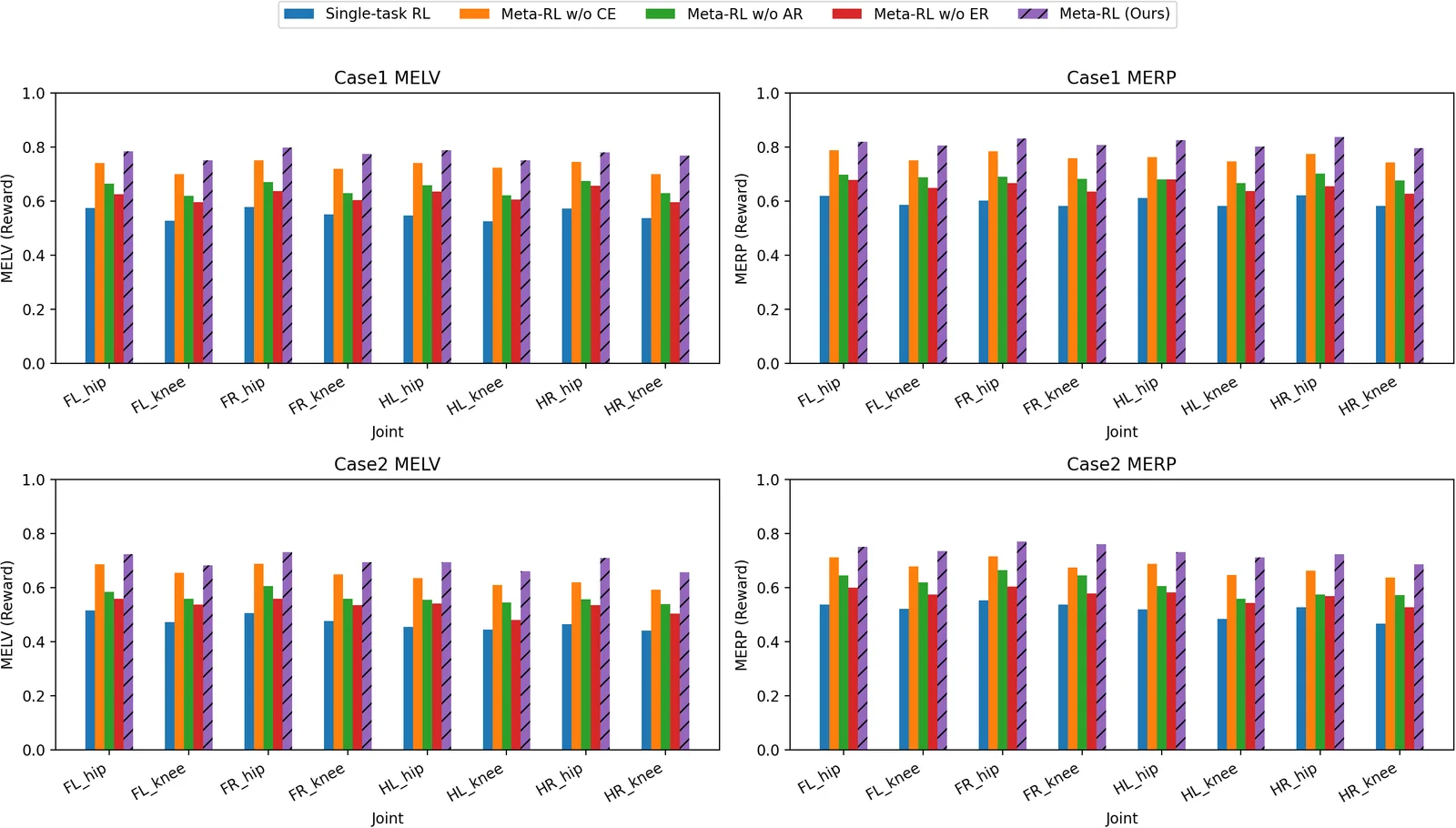
Ablation results for MELV and MERP under different configurations of locked joints. Higher values indicate better velocity tracking and posture regulation.

Removing the context encoder consistently lowers MELV and MERP, supporting the use of short interaction histories to infer the fault dynamics of each task. Removing the AR network causes a moderate decline under faults affecting a single joint and a larger decline under multiple joint faults, indicating the contribution of routing tailored to each task. Removing ER also lowers performance, particularly under severe faults, supporting its use during adaptation. The full ARER-MetaRL model achieves the highest MELV and MERP in most settings.

Fig 3 compares cumulative rewards across the ablation variants. The reward of Single-task RL drops sharply as fault severity increases. Removing any of the three components also reduces the reward relative to the full model. ARER-MetaRL maintains the highest cumulative reward, indicating that the components jointly improve adaptation to joint locking faults.

**Fig 3:**
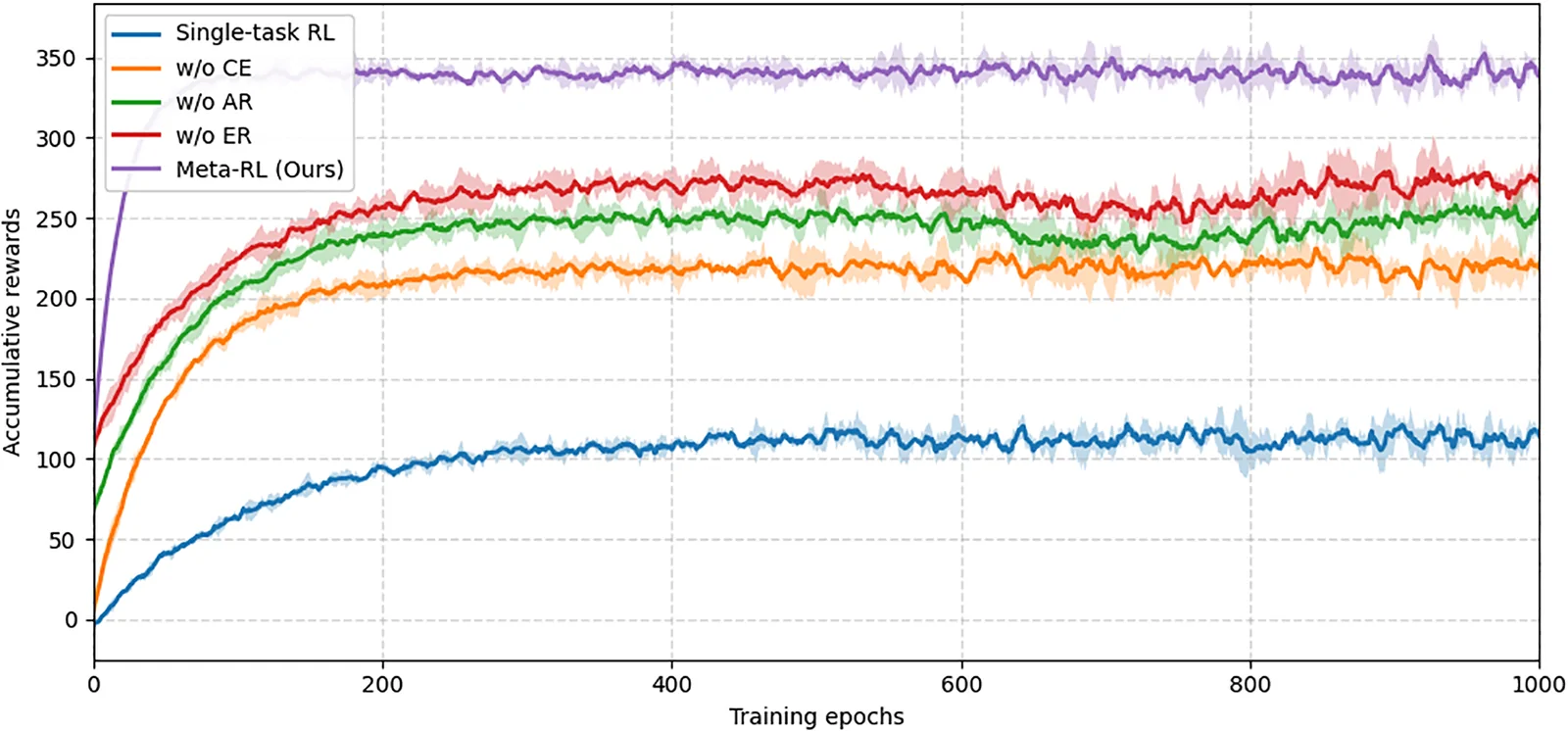
Cumulative rewards for the ablation variants as the severity of joint locking faults increases. Higher rewards indicate better performance under the combined locomotion objective.

Fig 4 shows representative foot trajectories for Single-task RL and ARER-MetaRL under joint locking faults. The baseline exhibits irregular oscillations and trajectory drift after fault onset, particularly when multiple joints are locked. ARER-MetaRL produces smoother, more repeatable trajectories that remain closer to nominal locomotion patterns, indicating better preservation of gait coordination after adaptation.

**Fig 4:**
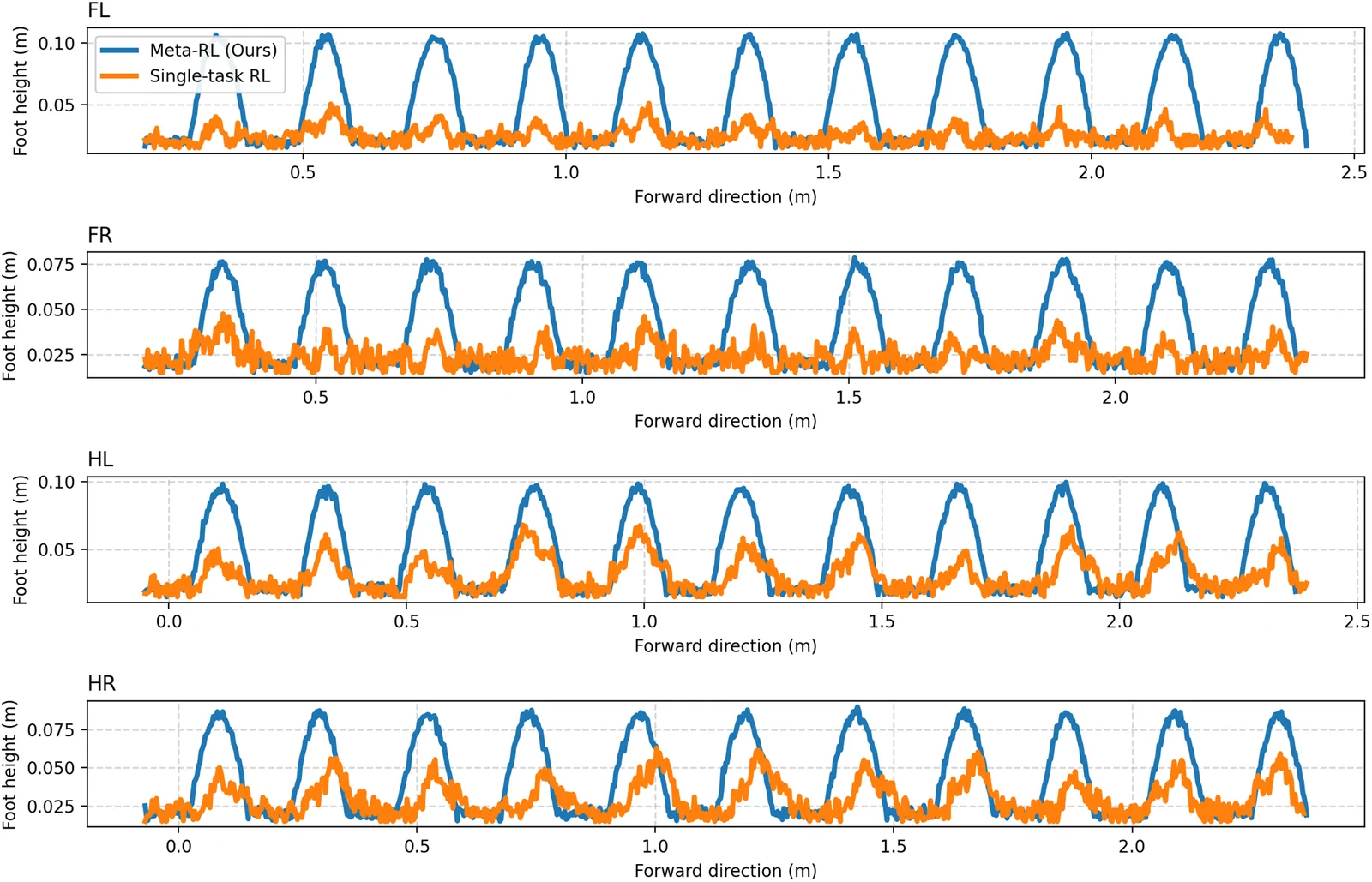
Representative foot trajectories under joint locking faults. ARER-MetaRL produces smoother, more repeatable trajectories than Single-task RL.

### 4.3. Comparison with representative baselines

Fig 5 compares ARER-MetaRL with representative model-based and model-free baselines: FADM [29], GrBAL [30], Decision Transformer [31], REDQ [32], and Meta-RL based on context with RAG [18]. Performance is assessed using MELV and MERP under sampled configurations of locked joints.

**Fig 5:**
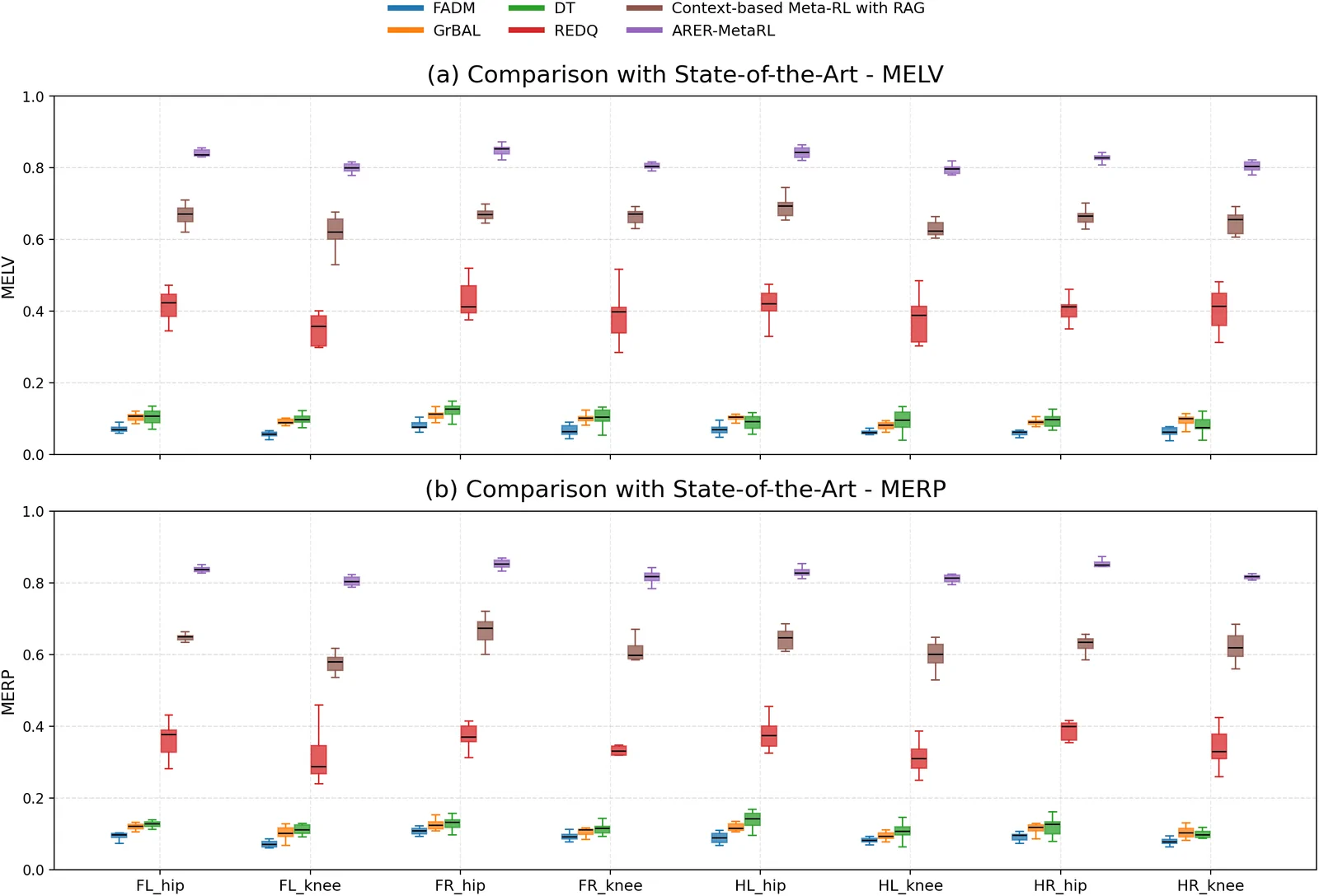
Comparison of locomotion performance under sampled configurations of locked joints. MELV measures velocity tracking, and MERP measures roll–pitch regulation. Higher values indicate better performance.

ARER-MetaRL achieves the highest MELV and MERP across the evaluated joint configurations, indicating better forward velocity tracking and roll–pitch regulation. Its advantage over Meta-RL based on context with RAG is modest but consistent and is particularly apparent for faults in knee joints, which strongly constrain gait dynamics. Together with the ablation results, these comparisons support the contributions of AR routing and ER to adaptation across the evaluated fault and terrain conditions.

### 4.4. Evaluation across motion and terrain conditions

To evaluate robustness beyond nominal forward locomotion, we test diagonal motion and locomotion on rough terrain under sampled configurations of locked joints. Case One and Case Two are representative fault configurations drawn from the evaluation distribution. Case One is a relatively mild fault affecting a single joint, whereas Case Two is a more severe configuration with different joint locations and stuck angles. Both cases are used in the evaluations of diagonal motion and locomotion on rough terrain.

Fig 6 shows representative trajectories during diagonal motion. The trajectory labeled “No Disabled” provides a fault-free reference with regular, repeatable cycles. ARER-MetaRL closely follows this reference, preserving the nominal motion pattern despite the joint constraints. Single-task RL exhibits larger deviations and oscillatory drift under joint locking faults, indicating poorer motion regulation during diagonal locomotion.

**Fig 6:**
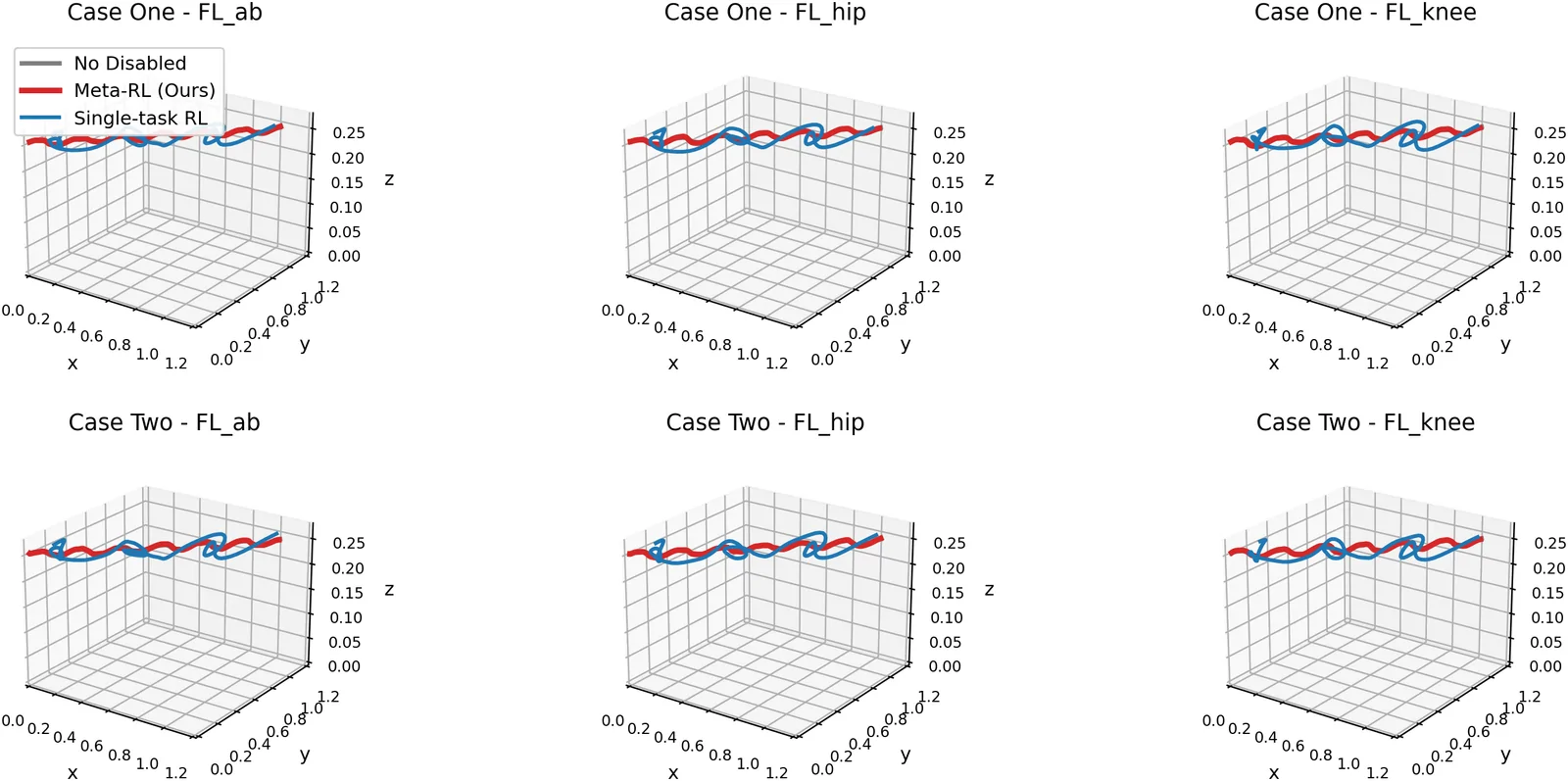
Representative trajectories during diagonal motion in Case One and Case Two under joint locking faults. **No Disabled** denotes the fault-free reference with regular periodic motion. **ARER-MetaRL** closely follows the reference, while Single-task RL exhibits larger deviations.

Fig 7 reports MELV and MERP for the diagonal motion task under different configurations of locked joints. ARER-MetaRL achieves higher values than Single-task RL in both Case One and Case Two, indicating better velocity tracking and posture regulation under these faults.

**Fig 7:**
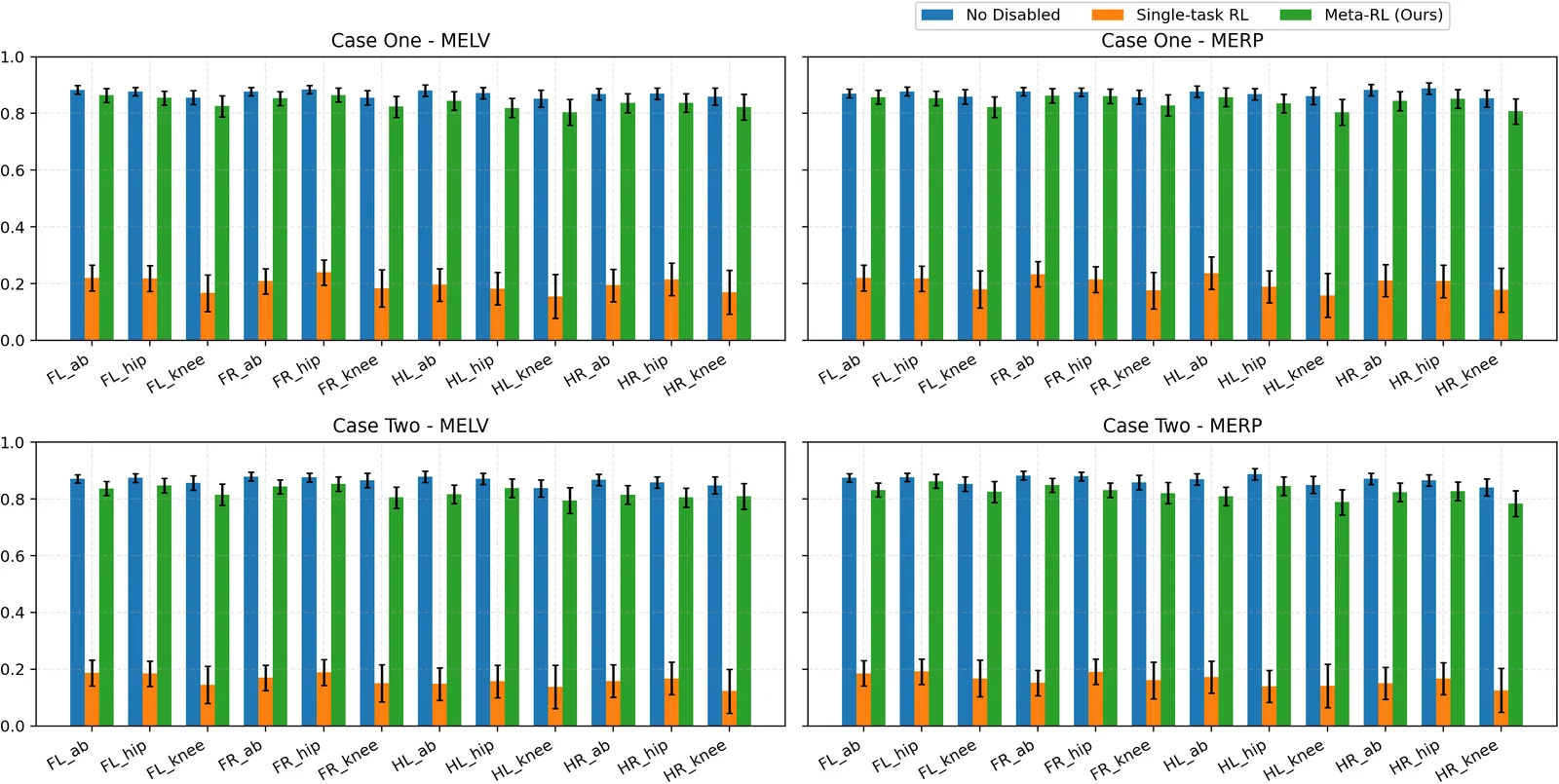
MELV and MERP during diagonal motion in Case One and Case Two under joint locking faults. Higher values indicate better velocity tracking and posture regulation. ARER-MetaRL consistently achieves higher rewards than Single-task RL.

Fig 8 shows trajectories during locomotion on rough terrain. The fault-free reference, labeled “No Disabled”, retains a periodic pattern despite terrain disturbances. ARER-MetaRL remains close to this reference under joint locking faults, indicating effective gait regulation on uneven terrain. Single-task RL exhibits greater drift and larger deviations from the reference.

**Fig 8:**
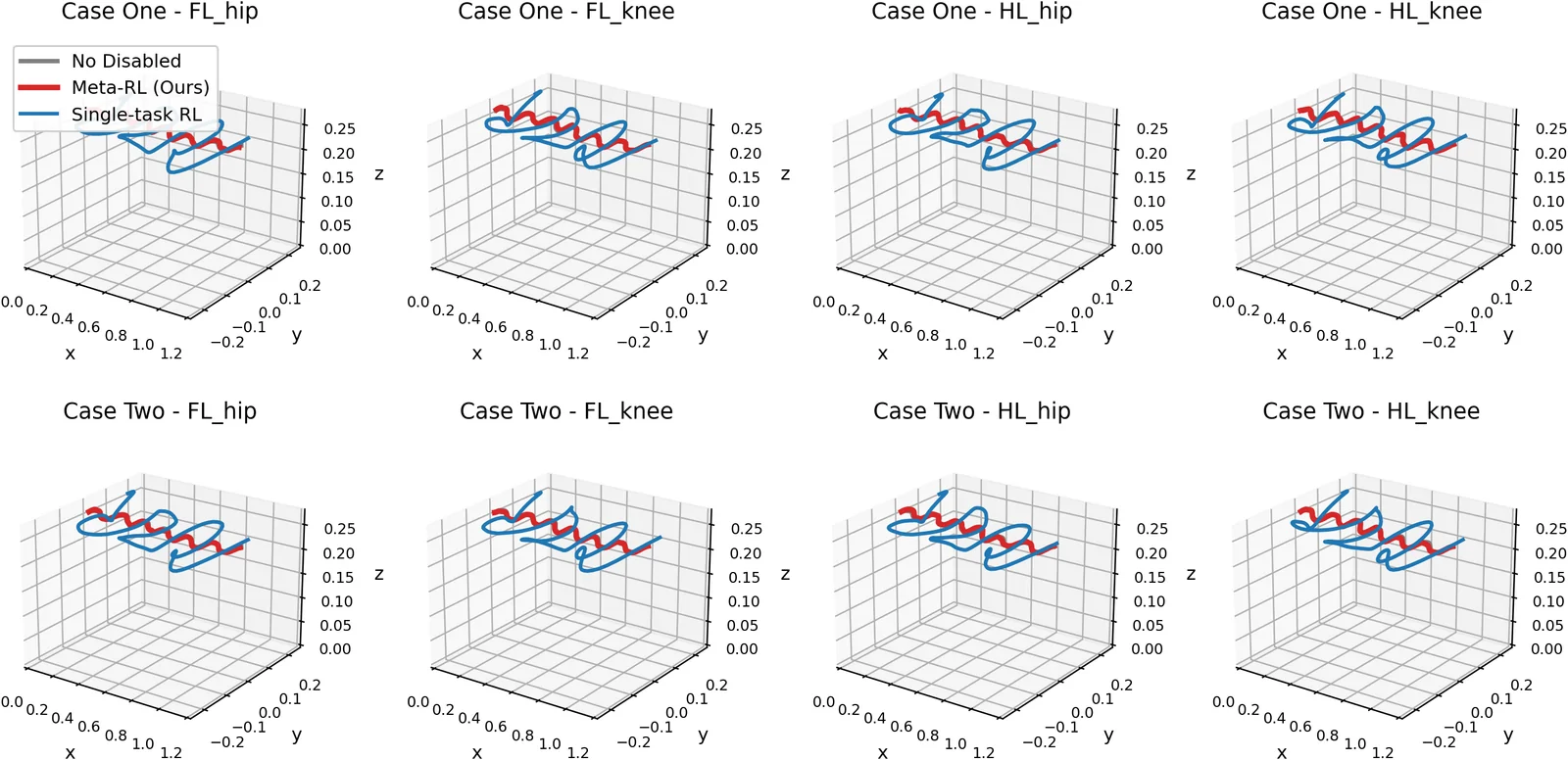
Representative trajectories during locomotion on rough terrain in Case One and Case Two under joint locking faults. **ARER-MetaRL** remains close to the No Disabled reference, while Single-task RL exhibits greater drift and larger deviations.

Fig 9 shows that ARER-MetaRL achieves higher MELV and MERP than Single-task RL on rough terrain, indicating better velocity tracking and posture regulation in these conditions.

**Fig 9:**
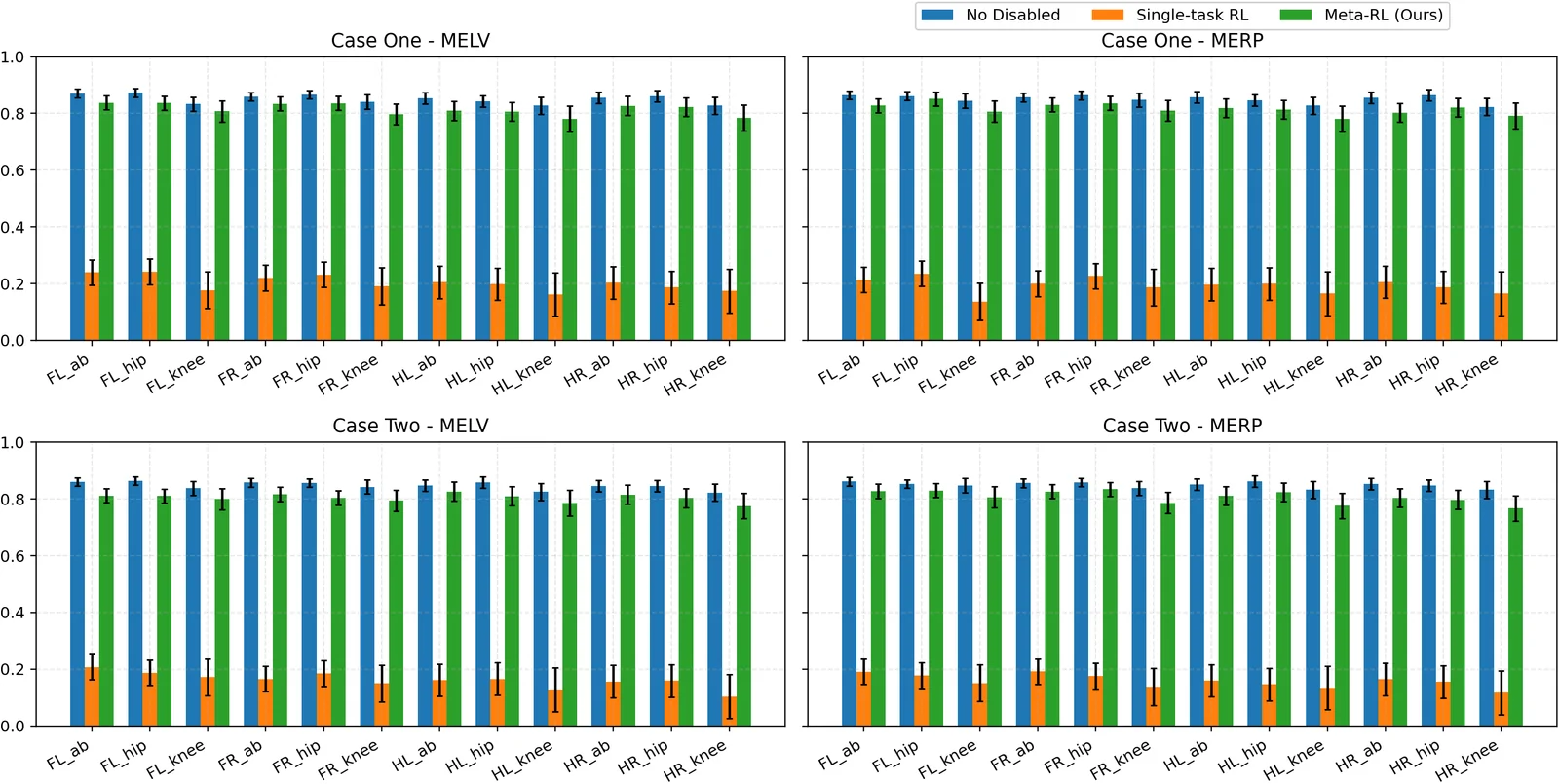
MELV and MERP during locomotion on rough terrain in Case One and Case Two under joint locking faults. Higher values indicate better velocity tracking and posture regulation. ARER-MetaRL, labeled Meta-RL (Ours), achieves higher rewards than Single-task RL.

To assess the effect of fault severity, we vary the number of simultaneously stuck joints and evaluate MELV and MERP. Fig 10 shows that performance declines as more joints become unavailable for control. ARER-MetaRL maintains substantially higher rewards than Single-task RL at every severity level, indicating better velocity tracking and posture regulation under increasingly severe faults.

**Fig 10:**
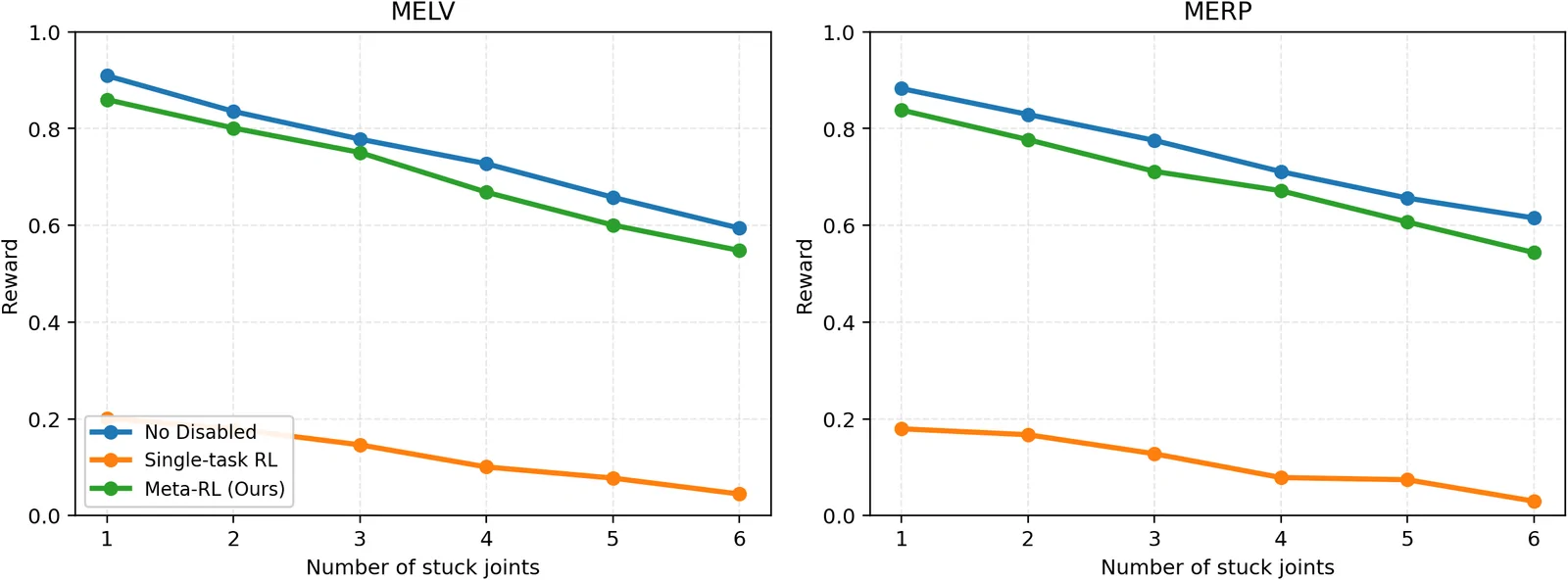
Performance versus the number of concurrently stuck joints. Both MELV and MERP decrease as fault severity increases. ARER-MetaRL consistently maintains higher rewards than Single-task RL across all severity levels.

Table 5 lists the mean values plotted in Fig 10. The performance gap remains substantial across the evaluated severity levels. With one stuck joint, ARER-MetaRL achieves MELV/MERP values of 0.86/0.84, compared with 0.21/0.18 for Single-task RL. With six simultaneously stuck joints, the values are 0.55/0.54 for ARER-MetaRL and 0.05/0.03 for Single-task RL. Both metrics are normalized rewards for velocity tracking and roll–pitch regulation.

**Table 5:** Mean values from the study of fault severity in Fig 10, rounded to two decimal places. The fault-free reference is labeled “No Disabled” in the plot.

$N_{f}$	MELV			MERP		
	Reference	Single-task	ARER-MetaRL	Reference	Single-task	ARER-MetaRL
1	0.91	0.21	0.86	0.88	0.18	0.84
2	0.83	0.18	0.80	0.83	0.17	0.78
3	0.78	0.15	0.75	0.77	0.13	0.71
4	0.73	0.10	0.67	0.71	0.08	0.67
5	0.66	0.08	0.60	0.66	0.07	0.61
6	0.59	0.05	0.55	0.62	0.03	0.54

We also evaluate combinations of two stuck joints to assess performance beyond faults affecting a single joint. Fig 11 presents heatmaps of MELV and MERP, each divided along the diagonal: the *upper-right* triangle shows ARER-MetaRL, and the *lower-left* triangle shows Single-task RL. Each off-diagonal cell represents a configuration of two faulty joints, with higher values indicating better tracking and posture regulation. ARER-MetaRL achieves higher rewards for most joint pairs, demonstrating improved performance when two joints are constrained simultaneously.

**Fig 11:**
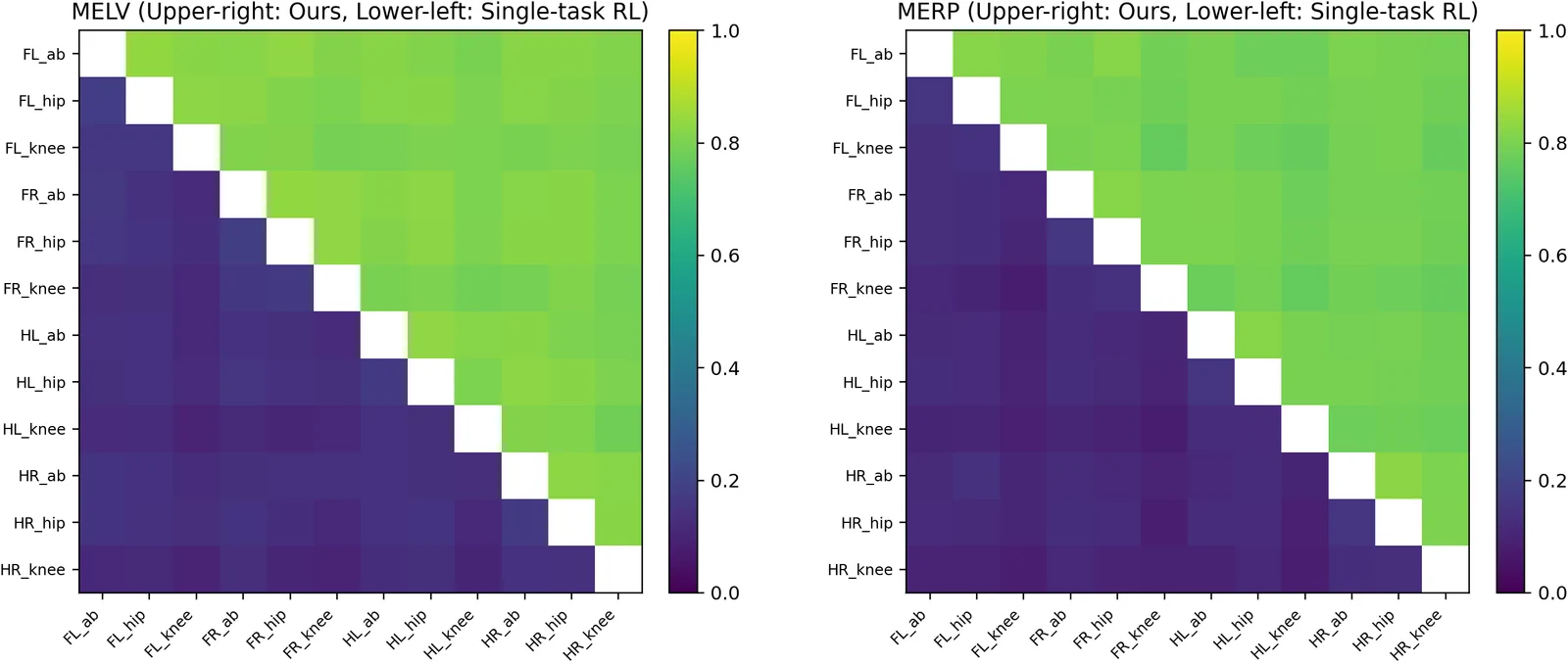
MELV and MERP for configurations with two stuck joints, shown as heatmaps divided along the diagonal. Each off-diagonal cell represents one configuration of two faulty joints. The *upper-right* triangle shows ARER-MetaRL, and the *lower-left* triangle shows Single-task RL. Higher values indicate better velocity tracking and posture regulation.

### 4.5. Practical deployment considerations

The controlled evaluation provides a consistent basis for comparing adaptation across joint locations, stuck angles, numbers of simultaneous faults, and terrain conditions. Transfer to a physical quadruped will require accounting for actuator delay and bandwidth, torque saturation, gearbox backlash, sensor noise, communication latency, uncertainty in foot–ground contact, and differences in mass and inertia. These factors can affect both the context inferred from short rollouts and the stability of online gradient updates.

Future hardware evaluation will combine actuator identification, torque and latency modeling, randomization of sensor noise and contact conditions, and progressive tests on a tethered or supported quadruped. Testing will begin with one locked joint before introducing simultaneous faults. Performance will be quantified using velocity tracking error, the root mean square errors in roll and pitch, recovery time, fall rate, actuator energy consumption, and adaptation time. The framework will also be extended to power loss, reduced actuator torque, intermittent actuation, and freely swinging joints.

## 5. Conclusion

This study presented ARER-MetaRL for quadruped locomotion under joint locking faults. The framework combines task inference from context, modular routing tailored to each task through AR, and fast adaptation regularized by ER. Short interaction histories provide task information for routing, while gradient smoothing and meta-updates weighted by fitness support adaptation using limited data.

Experiments in Isaac Sim show that ARER-MetaRL achieves higher MELV and MERP than Single-task RL, the ablated variants, and the evaluated baselines, as well as higher cumulative rewards in the ablation study. It also outperforms Single-task RL in tests of diagonal motion, rough terrain, simultaneous faults, and fault severities excluded from training. As the number of stuck joints increases from one to six, its MELV/MERP values decrease from 0.86/0.84 to 0.55/0.54, compared with a decline from 0.21/0.18 to 0.05/0.03 for Single-task RL. These results demonstrate improved adaptation across heterogeneous configurations of locked joints.

Future research will extend the task distribution to complete power loss, reduced actuator torque, intermittent failures, and freely swinging joints. Transfer from simulation to reality will incorporate actuator identification, modeling of delays and torque limits, randomization of sensor and contact conditions, and safety constraints on online adaptation. Physical robot experiments will measure velocity tracking error, roll and pitch stability, recovery time, fall rate, energy consumption, and adaptation latency as faults are progressively introduced. Multimodal fault inference will also combine proprioception with motor current, temperature, and contact information to improve fault identification and enable timely switching to a safe fallback controller.

## Abbreviations

AR: Adjudicate Reconfiguration
ARER-MetaRL: Adjudicate Reconfiguration and Evolving Regularization Meta Reinforcement Learning
CNN: Convolutional Neural Network
CPG: Central Pattern Generator
DT: Decision Transformer
ER: Evolving Gradient Regularization
ESO: Extended State Observer
FADM: Fast Adaptation Dynamics Model
FTC: Fault-Tolerant Control
GAE: Generalized Advantage Estimation
GrBAL: Gradient-Based Adaptive Learner
GRU: Gated Recurrent Unit
HAA: Hip Abduction/Adduction
HFE: Hip Flexion/Extension
KFE: Knee Flexion/Extension
LADRC: Linear Active Disturbance Rejection Control
MDP: Markov Decision Process
MELV: Mean Episode Linear Velocity Tracking Reward
MERP: Mean Episode Roll–Pitch Tracking Reward
Meta-RL: Meta Reinforcement Learning
OOD: Out-of-Distribution
PD: Proportional–Derivative
PPO: Proximal Policy Optimization
RAG: Reference Action Generator
REDQ: Randomized Ensembled Double Q-Learning
RL: Reinforcement Learning
SAC: Soft Actor–Critic
